# Similarity and diversity of the tumor microenvironment in multiple metastases: critical implications for overall and progression-free survival of high-grade serous ovarian cancer

**DOI:** 10.18632/oncotarget.12106

**Published:** 2016-09-19

**Authors:** Andreas Heindl, Chunyan Lan, Daniel Nava Rodrigues, Konrad Koelble, Yinyin Yuan

**Affiliations:** ^1^ Centre for Evolution and Cancer, The Institute of Cancer Research, London, UK; ^2^ Centre for Molecular Pathology, Royal Marsden Hospital, London, UK; ^3^ Division of Molecular Pathology, The Institute of Cancer Research, London, UK; ^4^ Department of Gynecologic Oncology, Sun Yat-sen University Cancer Centre, Guangzhou, China; ^5^ State Key Laboratory of Oncology in South China, Collaborative Innovation Centre for Cancer Medicine, Guangzhou, China; ^6^ Division of Cancer Therapeutics, The Institute of Cancer Research, London, UK; ^7^ Department of Histopathology, Royal Marsden Hospital, London, UK

**Keywords:** high-grade serous ovarian cancer, locally advanced disease, tumor microenvironment, automated image analysis, ecological diversity

## Abstract

The tumor microenvironment is pivotal in influencing cancer progression and metastasis. Different cells co-exist with high spatial diversity within a patient, yet their combinatorial effects are poorly understood. We investigate the similarity of the tumor microenvironment of 192 local metastatic lesions in 61 ovarian cancer patients. An ecologically inspired measure of microenvironmental diversity derived from multiple metastasis sites is correlated with clinicopathological characteristics and prognostic outcome. We demonstrate a high accuracy of our automated analysis across multiple sites. A low level of similarity in microenvironmental composition is observed between ovary tumor and corresponding local metastases (stromal ratio *r* = 0.30, lymphocyte ratio *r* = 0.37). We identify a new measure of microenvironmental diversity derived from Shannon entropy that is highly predictive of poor overall (*p* = 0.002, HR = 3.18, 95% CI = 1.51-6.68) and progression-free survival (*p* = 0.0036, HR = 2.83, 95% CI = 1.41-5.7), independent of and stronger than clinical variables, subtype stratifications based on single cell types alone and number of sites. Although stromal influence in ovary tumors is known to have significant clinical implications, our findings reveal an even stronger impact orchestrated by diverse cell types. Quantitative histology-based measures can further enable objective selection of patients who are in urgent need of new therapeutic strategies such as combinatorial treatments targeting heterogeneous tumor microenvironment.

## INTRODUCTION

Ovarian cancer is the most fatal gynecological malignancy [19, 36]. Approximately 75% of patients have metastatic diseases at diagnosis [15], and survival rate for International Federation of Gynecology and Obstetrics (FIGO) stage III-IV ovarian cancer ranges from 25% to 37% [43]. The vast majority (70-80%) of ovarian cancer deaths are from high-grade serous carcinoma (HGSOC) with hardly any change in overall survival in the past years [6]. This is partly due to the distinctive biology of HGSOC: the lack of an anatomical barrier in the peritoneal cavity to cancer cell dissemination likely originated from the fallopian tube or precursor cells in the ovary [6, 22, 24, 27, 37, 39].

To control this highly metastatic disease, concerted efforts have led to a new understanding of genetic heterogeneity in ovarian cancer and developments of new targeted therapies and chemotherapy agents [2, 4, 5, 18, 30, 33, 48, 49]. However, responses to therapies are often short-lived, and patients eventually relapse [51]. Understanding the contribution of the microenvironment to ovarian cancer progression will lead to the development of new biomarkers and provide the basis of improved therapeutic efficiency [4, 20, 29]. The desire to understand cancer-microenvironmental interactions has fuelled a developing interest in studying cancer from a novel perspective: ecology [3, 17, 23, 32, 40]. Seeing cancer cells as a ‘species’ disrupting the homeostasis of a complex ecosystems rather than just as a group of mutated cells [3] allows for drawing analogies to ecological hypothesis such as that complete eradication of a species is most likely impossible due to their ability to evolve adaptive strategies [17]. Another prominent example inspired by ecology is the “seed and soil” theory saying that in order to metastasize, the soil (microenvironment at the metastasis site) is as important as the seed (malignant cell) [34, 41]. Understanding and early interventions of the primary “seeds” and metastatic “soil” are required for better clinical management of cancer. Within a Darwinian framework, such analyses can reveal distinct microenvironments, or, the habitats of cancer, that can employ a number of different ecological forces driving tumor development and spread [14]. Studies of these ecological forces occurring in tumors can benefit from application of statistical tools routinely used in ecological studies. Histology samples provide an abundance of data as input for these methods due to preserved spatial context. Thus, spatial analysis empowered by large-scale analysis of archival histology samples could facilitate studies of ecological interactions in human tumors with far-reaching implications [23, 32].

Our previous work [25] demonstrated how fully automated image analysis using hematoxylin and eosin (H&E) slides of ovary tumors can enable the identification of ovarian cancer subtypes that were consistent with molecular subtyping previously reported [26, 48]. These subtypes were defined based on proportions of cells in histological ovary tumor sections alone: a high lymphocyte ratio group with good prognosis and a high stromal ratio group with poor prognosis [25]. However, different types of cells interact and co-exist with high spatial heterogeneity within a patient, yet the collective effect of cell diversity has not been studied. Besides a recent paper [12] where the authors observed large variations in intra-case comparisons of stromal characteristics between ovary and metastases, the microenvironment of tumors other than those from the ovary has received little investigation. In this paper, we apply ecologically inspired diversity measurements based on Shannon entropy to assess the heterogeneity of microenvironments in multiple local metastasis sites of HGSOC, including ovary, omentum, peritoneum, lymph node and appendix. Our aims are: 1) to establish the use of automated histology analysis for studying multiple metastasis sites besides ovary tumors, 2) to evaluate the similarity and ecological diversity of the microenvironment in these local metastases, 3) to understand the clinical implication of microenvironmental characteristics in HGSOC metastases.

## RESULTS

### Automated analysis of tumor microenvironment in local HGSOC metastases

**Figure 1:**
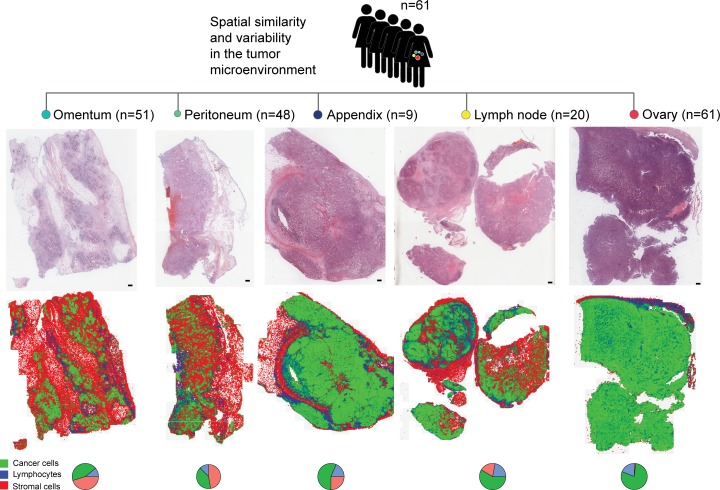
Study design depicting automated analysis of tumor microenvironments in multiple metastases of 61 patients with locally advanced HGSOC H&E images are depicted next to spatial maps of cancer cell (green), lymphocyte (blue) and stromal cell (red) distributions. Pie charts depict the quantitative cell composition in respective sections. Scale bar represents 2 mm.

**Figure 2:**
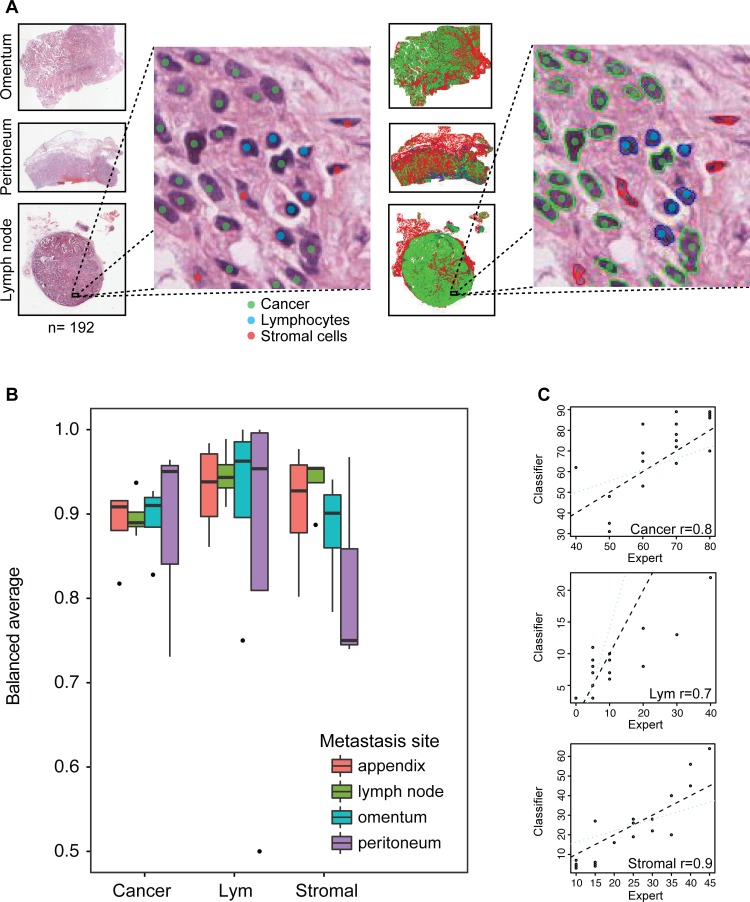
Histology image analysis and validation **A.** Illustration of our procedure collecting single-cell annotations from pathologists as colored dots (left) and superimposed automated cell classifications as colored contours (right). **B.** Boxplots depicting the balanced average (mean of sensitivity and specificity) for all three cell classes computed from expert annotations and automated cell classifications of 4,633 cells in total. The horizontal line represents the median. The whiskers show the highest value respectively lowest value that is within 1.5-fold inter-quartile range. **C.** Spearman correlation analysis of cell ratio scores of expert pathologist and automated classification.

We previously reported a high accuracy of our automated histological analysis system in identifying three major cell types (cancer cells, lymphocytes and stromal cells) in whole-section H&E histology samples of ovary tumors [25]. This system enabled the identification of ovarian cancer subtypes that were consistent with molecular subtyping previously reported [26, 48]. These subtypes were defined based on proportions of cells alone in the ovary tumors: a high lymphocyte ratio group with good prognosis and a high stromal ratio group with poor prognosis [25]. Here, we evaluated the accuracy of our system in analyzing H&E sections of tumors from other local metastases including omentum, peritoneum, appendix, spleen, umbilicus and lymph node using two approaches (Figure 1, Methods). First, we tested the accuracy of single-cell classification using a collection of 4,633 single cells annotated by two pathologists that were not used to train the classifier (DNR and KK, Methods, Figure 2a). We found a good overall performance of our classifier compared with the pathologists’ annotations with high mean balanced sensitivity and specificity scores across sites (cancer cells 0.90, lymphocytes 0.82 and stromal cells 0.89, Figure 2b). Stromal cell classification in peritoneum has the lowest balanced average measure due to a higher frequency of tissue contraction artifacts as tissue gaps originated from formalin fixation and paraffin embedding procedures. Secondly, we measured the correlation between automated and pathologist's cell proportion scores and observed a good level of correlation between automated and pathologist's scores of cell proportions (cancer: *r* = 0.8, lymphocytes: *r* = 0.7, stromal cells: *r* = 0.9, Figure 2c). The presence of a small amount of normal epithelial cells in the appendix was responsible for misclassification as cancer cells, resulting in a lower correlation with the pathologist's cancer cell proportion score. However, we observed in general a low amount of normal epithelial cells and the overall accuracy of cancer cell classification is high (median = 87%-96%, Figure 2b). We subsequently applied this automated image analysis tool on 192 local tumor lesions from 61 patients (Methods, Table 1).

**Table 1:** Patient sample characteristics (*n* = 61) stratified by the MetDiv score or stromal ratio

Factor	Distribution		*P*	Distribution		*P*
	High MetDiv	Low MetDiv		Stromal high	Stromal low	
**Number**	11	50		36	25	
**Age**			**0.793**			**0.73**
Median	55	55		55	55	
Range	(43-75)	(22-82)		(22-75)	(36-82)	
**Death**			**0.084**			**0.167**
No	1 (9.1%)	19 (38%)		9 (25%)	11 (44%)	
Yes	10 (90.9%)	31 (62%)		27 (75%)	14 (56%)	
**Recurrence**			**0.084**			**0.706**
Yes	11 (100%)	8 (16%)		32 (89%)	21 (84%)	
No	0 (0%)	42 (84%)		4 (11%)	4 (16%)	
**FIGO stage**			**1**			**0.682**
IIIc	10 (90%)	45 (90%)	9.1×10^−7^**	33 (92%)	22 (88%)	0.80
IV	1 (10%)	5 (10%)	0.14	3 (8%)	3 (12%)	0.13
**Debulking**			**1**			**0.99**
Optimal	6 (54%)	25 (50%)		16 (44%)	15 (60%)	
Suboptimal	5 (46%)	25 (50%)		20 (56%)	10 (40%)	
**Primary chemotherapy regimen**			0.002*			**0.69**
TC/TP †	11 (100%)	43 (86%)		31 (86%)	23 (92%)	
CBP ‡	0 (0%)	7 (14%)		5 (14%)	2 (8%)	

Numbers are mean (range). Kruskal-Wallis test was used for testing association between a continuous variable and a categorical variable. Fisher's exact test was used to test for association between categorical variables.

† TC/TP: Paclitaxel/docetaxel plus carboplatin/cisplatin

‡ CBP: Cyclophosphamide, bleomycin plus carboplatin

### Comparison of microenvironmental composition between ovary tumors and other local metastasis sites

**Figure 3:**
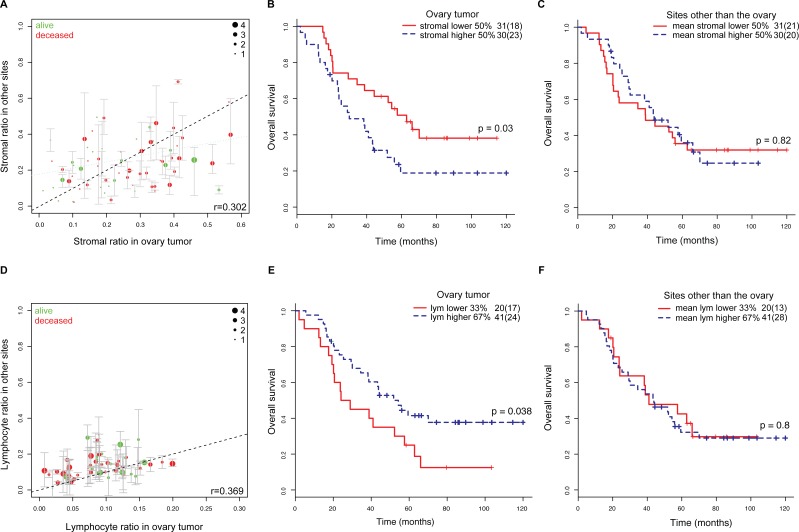
Comparison of tumor microenvironments in multiple ovarian metastases **A.** Comparison of the distribution of stromal cells in ovary tumor and other corresponding metastasis sections in the same patients. Green: patients alive after 10 years from surgery, red: deceased patients. The diameter of the circle indicates the amount of metastasis sites. Whiskers show min max cell ratios of the metastases. **B.** Overall survival stratified by stromal ratio of the ovary tumor (red: stromal ratio < 0.24, blue: stromal ratio≥0.24). **C.** Overall survival stratified by the mean stromal ratio of all metastasis sites excluding ovary (red: mean stromal ratio < 0.2, blue: mean stromal ratio≥0.2). **D.** Distribution of lymphocytes in ovary and other corresponding metastasis sections in the same patients. Color-coding and circle diameter as in A. **E.** Overall survival stratified by lymphocyte ratio of the ovary tumor (red: lym ratio < 0.07; blue: lym ratio≥0.07). **F.** Overall survival stratified by the mean lymphocyte ratio of all metastasis sites excluding ovary (red: mean lym ratio < 0.1; blue: mean lym ratio≥0.1).

Based on the histology image analysis results, we first compared the cellular composition of ovary tumors by examining individual cell types. We found a low correlation between stromal ratio measured in the ovary and mean stromal ratio in other local metastasis sites of the same patient (Pearson correlation *r* = 0.302, *p* = 0.03, Figure 3a, Methods). Consistent with our previous study [25], the high stromal ratio group, defined by dividing patients into two equal-size groups based on the ratio of stromal cells to all cells in ovary tumors, was associated with poor overall survival (*p* = 0.03, HR = 1.99, 95% CI = 1.07-3.72, Figure 3b; Supplementary Figure 1b) However, stromal ratio defined in any local site other than the ovary, or the mean stromal ratio of those sites were not prognostic (*p* > 0.05, Table 3, Figure 3c). A moderate correlation between lymphocyte ratio measured in ovary tumor and mean lymphocyte ratio in other metastasis sites of the same patient was also observed (Pearson correlation *r* = 0.369, *p* = 0.003, Figure 3d). Low lymphocyte ratio was associated with poor overall survival (*p* = 0.04, HR = 0.52, 95% CI = 0.28-0.97, Figure 3e; Supplementary Figure 1a). Neither lymphocyte ratio defined in any local site other than the ovary nor the mean lymphocyte ratio of these sites was prognostic (*p* > 0.05, Table 3, Figure 3f).

**Table 2:** Metastases sample distribution (*n* = 131) stratified by the MetDiv score and stromal ratio

Factor	Distribution		*P*	Distribution		*P*
	High Metdiv	Low MetDiv		Stromal high	Stromal low	
**Number**	33	78		108	23	
**Metastasis sites**			0.13			0.57
Appendix	4 (3%)	5 (4%)		8 (6%)	1 (1%)	
Lymph node	1 (1%)	19 (15%)		3 (2%)	17 (13%)	
Omentum	11 (8%)	40 (31%)		26 (20%)	25 (19%)	
Peritoneum	13 (10%)	35 (27%)		23 (18%)	25 (19%)	
Spleen	0 (0 %)	1 (1%)		1 (1%)	0 (0 %)	
Umbilicus	0 (0 %)	2 (2%)		2 (2%)	0 (0 %)	

Kruskal-Wallis test was used for statistical testing.

### Tumor microenvironmental diversity of ovarian local metastases is associated with poor overall and progression-free survival

To measure the collective characteristics of the microenvironment, we next examined tumor diversity measures that consider all cell types simultaneously. The Shannon diversity index that measures species diversity in an ecosystem was calculated for each tumor (Methods). A high score indicates diverse cell types with similar proportions, whilst a low score suggests dominance of one cell type. We first compared the distribution of Shannon diversity for each tumor site. Appendix metastasis has significantly higher level of diversity compared with lymph node metastasis (Kruskal-Wallis test *p* = 0.008, Supplementary Figure 2a). Analysis of averaged cell ratios in these sites showed that lymph node has in general a higher proportion of lymphocytes, as expected (Supplementary Figure 2b). The appendix, on the other hand, has higher proportions of stromal cells and lymphocytes, which explained the high diversity score.

**Figure 4:**
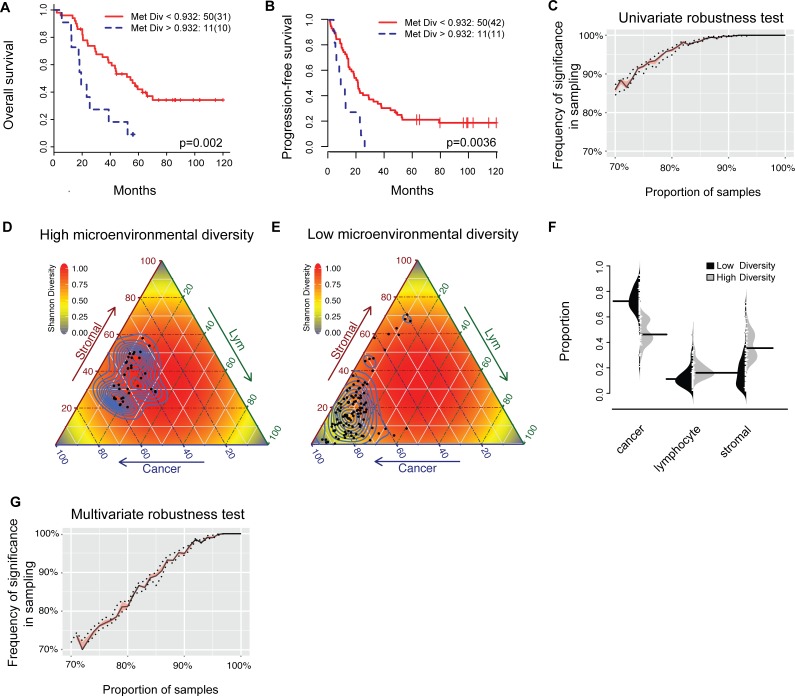
Prognostic value of the MetDiv score **A.** and **B.** Kaplan-Meier survival curves to illustrate duration of OS and PFS for patients with a low (red) or high (blue) MetDiv score. **C.** Random sampling to test for robustness of MetDiv in univariate survival analysis. **D.** and **E.** Visualization of the microenvironmental composition for individual metastases for patients with low and high MetDiv score. Each black point in the triangle plot is a metastasis and background is colored by the theoretical distribution of Shannon diversity. **F.** Bean plot depicting the distribution of cell ratios in patient groups defined by the MetDiv score. The black horizontal line in each distribution shows the median. **G.** Random sampling to test for robustness of MetDiv in multivariate survival analysis including MetDiv, Age, Stromal ratio and Lym ratio measured in the ovary tumor.

To measure the level of microenvironmental diversity for each patient, we calculated the mean of diversity scores in all tumors other than those from the ovary (MetDiv). By relating it to survival, we found that a high MetDiv score was associated with poor OS (*p* = 0.002, hazard ratio (HR) = 3.18, 95% confidence interval (CI) = 1.51-6.68, Figure 4a; Supplementary Figure 1c) and PFS (*p* = 0.0036, HR = 2.83, 95% CI = 1.41-5.7, Figure 4b, Table 3; Supplementary Figure 1d). Patients with diverse microenvironments in metastases have 9% 5-year OS and 0% PFS compared with 42% and 20% respectively for patients with more homogenous microenvironments. The stability of these survival analyses was evaluated by random sampling of progressively fewer samples from 100% (*n* = 61) to 70% (*n* = 42). Mean metastasis diversity remained prognostic over 85% of the time for univariate analysis (OS; Figure 4c). This confirms the stability of this score as a predictor for survival. In contrast, measures of mean diversity scores in all tumors including those from the ovary, microenvironmental diversity in ovary tumors, or variability within local metastases (standard deviation) were not found to be prognostic (Table 3, *p* > 0.05). In addition, we compared our scores with another widely used ecological diversity index, the Simpson index (Methods). A high correlation between the two indices (*r* = 0.98, Supplementary Figure 3, Methods) was observed, which is consistent with previous studies that found very little qualitative differences between these scores [28, 35]. We thus focused on the Shannon diversity index and MetDiv henceforth. Patient stratification based on MetDiv with respect to three cell types is illustrated in Figure 4d-4e. A shift in microenvironmental features from the low diversity group to the high diversity group is evident. Metastasis sites with low MetDiv scores contained a high amount of cancer cells, whereas those with highly diverse microenvironment shifted towards the center of the triangle plot due to a more diverse microenvironmental makeup.

**Table 3:** Prognostic value of MetDiv and other parameters in groups using progression-free and overall survival

Type	Variable	OS			PFS		
		HR (CI)	*p*	Conc	HR (CI)	*p*	Conc
All sites except ovary	**MetDiv**	3.18 (1.51-6.68)	0.002**	0.59	2.83 (1.41-5.7)	0.0036 **	0.57
Ovary-based parameters	Ovary Shannon Diversity	1.56 (0.18-13.8)	0.69	0.52	4.86 (0.68.46)	0.114	0.56
	Lym ratio in ovary	0.52 (0.28-0.97)	0.04*	0.57	0.81 (0.45-1.43)	0.48	0.53
	Stromal ratio in ovary	1.99 (1.07-3.72)	0.03*	0.59	1.66 (0.96-2.87)	0.067	0.59
Other site-specific parameters	Presence of omentum	1.5 (0.62-3.5)	0.39	0.54	2.03 (0.91-4.5)	0.083	0.56
	Stromal ratio in omentum	1.33 (0.68-2.59)	0.40	0.54	1.11 (0.62-1.98)	0.74	0.54
	Lym ratio in omentum	0.79 (0.41-1.54)	0.49	0.54	1.19 (0.66-2.14)	0.57	0.50
	Presence of peritoneum	0.79 (0.42-1.48)	0.46	0.55	0.61 (0.35-1.05)	0.076	0.57
	Stromal ratio in peritoneum	1.83 (0.82-4.07)	0.14	0.58	1.81 (0.87-3.77)	0.11	0.58
	Lym ratio in peritoneum	0.54 (0.24-1.21)	0.14	0.57	0.70 (0.34-1.43)	0.33	0.57
Clinical parameters	Age	2.76 (1.33-5.77)	0.0067 **	0.57	2.35 (1.22-4.53)	0.0104 *	0.56
	Chemo- therapy regime	1.018 (0.40-2.60)	0.97	0.50	0.72 (0.29-1.82)	0.491	0.508
	Debulking	1.57 (0.84-2.93)	0.159	0.52	1.30 (0.75-2.25)	0.344	0.507
	FIGO Staging	0.86 (0.26-2.79)	0.8	0.50	0.90 (0.36-2.28)	0.83	0.50
Other parameters	MetDiv including ovary	7.25 (0.34-1.54)	0.20	0.57	4.23 (0.74-24.05)	0.10	0.55
	SD of Shannon	1.00 (0.54-1.85)	0.99	0.50	0.79 (0.46-1.35)	0.38	0.52
	Number of sites	0.88 (0.65-1.21)	0.46	0.54	0.90 (0.68-1.18)	0.436	0.542
Multivariate analysis	**MetDiv** Age Stromal ratio in ovary Lym ratio in ovary	3.40 (1.59-7.27) 2.72 (1.27-5.83) 1.68 (0.88-3.21) 0.71 (0.38-1.34)	0.002 ** 0.01** 0.12 0.30	0.69	3.77 (1.77-8.03) 3.02 (1.48-6.12) 1.61 (0.91-2.82) 1.30 (0.71-2.41)	0.0006*** 0.0022** 0.10 0.39	0.65

Continuous variables were dichotomized into two groups using the optimal thresholding method. Analyses were univariate Cox regression unless stated otherwise. Conc: Concordance; HR: Hazard Ratio; CI: Confidence Interval.

* *p* < 0.05;

** *p* < 0.01;

*** *p* < 0.001.

Ovary Shannon Diversity is the Shannon Diversity measured in ovary tumors. SD of Shannon is the standard deviation computed over all metastases per patient. Lym (lymphocyte) / stromal ratio in ovary/omentum/peritoneum is the ratio of lymphocytes/stromal cells in all the cells presented in the respective sites.

### Metastasis diversity is not dominated by a cell type or metastatic site and remains stable with reduced amount of tissue

Subsequently, we tested whether the prognostic effect of our MetDiv score was driven by dominant cell types. When there is a dominant cell type, the Shannon diversity is low. Therefore a possible explanation of our observation is that a large amount of a single cell type in the metastases is responsible for a low diversity score and good prognosis. We then examined the distribution of cell types in the low and high MetDiv groups. We found no significant difference in the proportions of cancer cells, lymphocytes or stromal cells between the two groups (*p* > 0.1, Kruskal-Wallis test, Figure 4f). We next asked if MetDiv was driven by a specific metastasis site. MetDiv scores of these patients did not differ according to the presence of omentum, peritoneum, lymph node or appendix metastasis (*p* > 0.05, Supplementary Figure 4a-d). We next examined the distribution of MetDiv groups according to clinicopathologic parameters. MetDiv was not associated with FIGO stage, debulking status and age (*p* > 0.05) but chemotherapy regimen (*p* = 0.002, Table 1).

A factor that could influence the reliability of MetDiv score is the amount of tissue used for our analysis. To test this, we simulated the scenario that only half of a tumor was available and computed the MetDiv score based on the new data (Methods). We found that the difference between the original and the new Shannon diversity score was very small (standard deviation = 0.0016-0.0006; Supplementary Figure 5a). The prognostic value of MetDiv for OS and PFS based on the new scores remained significant (*p* < 0.005). We further evaluated the robustness of the MetDiv score by randomly sampling 100% / 75% / 50% of cells from the whole-section images (Supplementary Figure 5b), revealing an even smaller standard deviation of the diversity score (0.0003), thereby supporting the stability of the MetDiv score against variable amount of tissue used for analysis.

### Metastasis diversity is prognostic independent of clinical parameters

We tested the prognostic value of clinicopathologic and prognostic scores including age, debulking status, chemotherapy treatment regime, staging, and our previously reported subtyping based on immune/stromal ratio [25], and found that age (*p* = 0.00067, HR = 2.76, 95% CI = 1.33-5.77), stromal ratio in ovary tumors (*p* = 0.03, HR = 1.99, 95% CI = 1.07-3.72) and lymphocyte ratio in ovary tumors (*p* = 0.03, HR = 0.52, 95% CI = 0.28-0.97) were associated with OS (Table 3). For PFS, only age grouping (*p* = 0.01, HR = 2.35, 95% CI = 1.22-4.53: Table 3) was prognostic. In multivariate survival analysis with age, stromal and lymphocyte grouping, we found that the MetDiv score remains statistically significant (OS *p* = 0.002, HR = 3.40, 95% CI = 1.59-7.27; PFS *p* = 0.0006, HR = 3.77, 95% CI = 1.77-8.03, Table 3) followed by age (OS *p* = 0.01, HR = 2.72, 95% CI = 1.27-5.83; PFS p = 0.0022, HR = 3.02, 95% CI = 1.48-6.12). Stromal grouping and lymphocyte grouping were no longer significant (*p* > 0.05). The number of metastasis sites for each patient (OS *p* = 0.46, HR = 0.88 95% CI = 0.65-1.21, PFS p = 0.436, HR = 0.90 95% CI = 0.68-1.18) or the presence of omentum, peritoneum, lymph node, appendix metastasis was not correlated with OS or PFS (*p* > 0.5, Table 3). Random sampling was again performed for MetDiv in multivariate analysis of OS that include age, stromal and lymphocyte grouping (Methods). We found that with more than 80% (49) samples, more than 81.3% of the times MetDiv remained significant, supporting its stability as a prognostic marker (Figure 4g).

## DISCUSSION

Influences from stromal endothelial cells, immune regulation and cancer-associated fibroblasts were known to be important factors for tumor progression with roles in angiogenesis, inflammation and phenotype switching [1, 20, 52]. Prognostic subtypes directly related to immune or stromal components in ovary tumors have been consistently reported in large-scale studies of genomics [16, 33, 48, 49]. We have previously reported strong prognostic value of ovarian cancer subtypes based on the amount of immune or stromal infiltrate in ovarian tumor histological samples, where high amount of stromal cells was significantly associated with poor survival in contrast to lymphocytic infiltration which was found to correlate with favorable prognosis [25]. The most important finding in this study is that diverse microenvironment in multiple local metastases including omentum, peritoneal, lymph node and appendix is a novel feature of aggressive HGSOC. Its strong prognostic effect is independent of subtyping that consider a single cell type alone, microenvironmental diversity at a single tumor site, number of metastatic sites and standard clinical parameters in HGSOC. Therefore, our new study offers clear evidence that it is not just single cell type at play, but also that the collective contribution of diverse cells in the microenvironment parallels the acquisition of aggressive HGSOC phenotypes in locally advanced diseases.

Specifically, HGSOC patients with diverse microenvironment or high MetDiv score in their local metastases have a staggering 5-year overall survival of 9%, compared with 42% for patients with low MetDiv scores. Although there was a moderate degree of similarity in the amount of stromal cells between ovary tumors and other sites (Pearson correlation *r* = 0.302), only stromal cell abundance measured in ovary tumors but not in other sites was associated with poor overall survival in these locally advanced diseases (*p* = 0.03, HR = 1.99, CI = 1.07-3.72). Similar conclusions were drawn in our analysis comparing lymphocyte abundance in ovary and other sites. These results underscore the clinical significance of stromal and lymphocyte abundance in ovary tumors but not in other tumor sites.

The MetDiv score is, however, independent of and stronger than stromal or lymphocyte abundance in multivariate survival analysis (MetDiv *p* = 0.002, HR = 3.40 95% CI = 1.59-7.27, age *p* = 0.01, HR = 2.72 95% CI = 1.27-5.83, stromal ratio *p* = 0.12, HR = 1.68, 95% CI = 0.88-3.21 and lymphocyte ratio *p* = 0.30, HR = 0.71, 95% CI = 0.38-1.34). Debulking status is known as a key clinical variable for advanced ovarian cancer [7, 10, 50], and was found to be prognostic in our previous study that included patients with only ovary tumor samples (OS and PFS *p* < 0.01) [25]. However, it was not found to be associated with OS or PFS in the patient subset with more than one local metastasis under study here (*p* > 0.05, Table 3). Reasons for the lack of significance can be either the small sample size or the now dated definition of optimal debulking (residual disease no larger than 1 cm in maximum diameter).

Note that the MetDiv score is different from the ecosystem diversity index in our recent study of primary breast tumors [31]. Ovarian cancer is unique in its high metastatic potential due the lack of an anatomical barrier to cancer cell dissemination in the peritoneal cavity [5]. MetDiv thus represents an ecologically inspired measure that was specifically designed for the spatial analysis of HGSOC microenvironments, using a collection of 192 local metastasis tumors spreading across 61 patients. Despite variations in background of different tumor sites and variable numbers of metastasis sites per patient, we showed that MetDiv provides a generalizable measure of cell diversity across multiple sites, emphasizing the importance of considering the cumulative effects of diverse cell types at multiple locations that can contribute to HGSOC progression. Further studies based on our findings may suggest novel solution to the problem of therapeutic resistance in HGSOC management [44, 47] and lead to clinical innovations in treatment strategies. With recent advances in immunotherapy targeting immune checkpoints or cytokines, combinatorial therapies with these new agents and anti-VEGF drugs such as bevacizumab [9] to target multiple components may be carefully considered for patients with high MetDiv scores to mitigate the joint effect from multiple cell types. A further study to illuminate the complex network of cytokine and chemokine signaling between malignant cells and normal cells intertwined with growth factors underlying the prognostic effect of metastasis diversity will shed further light on this aspect.

In summary, we demonstrated how automated histology analysis coupled with ecological measures is effective in characterizing the metastatic microenvironment in locally advanced HGSOC, leading to clinically significant findings. Advantages of our proposed method are that it utilizes objective automated histology analysis, widely available paraffin-embedded specimens and standard H&E staining methods, and we demonstrated that it is robust against variable amount of tissue (> 50%) used for analysis. Limitations of our study include the lack of an independent test cohort, a two-dimensional representation of complex tumors, no spatial association measured between cell types, two different treatment regimens, only one histologic subtype and only basic cell types (stromal cells, lymphocytes and cancer cells). Furthermore the definition of optimal surgery was refined over time and changed from < 1 cm to minimal/none macroscopic residual disease [11]. Future efforts will focus on further validation of our automated system and the discrimination between different types of lymphocytes and stromal cells using immunohistochemistry approaches (e.g. fibroblasts, myofibroblasts, fibrocytes, vascular pericytes and endothelial cells) to further dissect the tumor microenvironment and consolidate their clinical relevance in HGSOC and other histologic subtypes as well as molecular profiling in independent cohorts with multiple sections per tumor.

Nevertheless, we expect that the need to understand the biological basis for coordinated influences from multiple microenvironmental cell types, and their concomitant effect on HGSOC treatment, will help shape future research on this devastating cancer type.

## MATERIALS AND METHODS

### Patient selection and characteristics

All patients with ovarian carcinoma, who received primary debulking surgery without neoadjuvant therapy between May 1999 and December 2010 at Sun Yat-sen University Cancer Centre, China, were reviewed. A total of 61 patients with International Federation of Gynecology and Obstetrics (FIGO) stage III-IV HGSOC ovarian cancer with at least one locally advanced resectable metastasis site were identified (Table 1). Resectable metastases were collected during debulking surgery. In total, 192 paraffin embedded blocks from 7 local metastasis sites (61 ovary, 51 omentum, 48 peritoneum, 9 appendix, 20 lymph node, 1 spleen, 2 umbilicus; Table 2) were obtained from 61 patients. For each patient 2-5 tumor sites were available (median *n* = 3). Patient consent and ethical approval were obtained by the institutional review board of Sun Yat-sen University Cancer Centre. Paraffin-embedded tumor blocks were retrieved from the hospital archive. Clinical data including clinicopathologic characteristics, treatment regimen and follow-up 10-year overall survival (OS) and progression-free survival (PFS) were collected (Table 1). OS was censored at the date of death or, for living patients, the date of last contact. PFS was censored at the date of death or progression, whichever occurred first, or the date of last contact for the patients alive and without recurrent disease. Progression was defined as serially rising CA125 levels, or any clinical or radiographic evidence of new lesions as either local/regional relapse or distant metastasis. Median OS and PFS were 43.33 months (range 1.97-120 months) and 19.8 months (0.97-120), respectively. All patients underwent debulking surgery that mainly consisted of total hysterectomy, salpingo-oophorectomy, omentectomy, nodal dissection, or intestinal surgery followed by chemotherapy. Optimal debulking was considered as residual disease no larger than 1 cm in maximum diameter evaluated by surgeons at surgery [8, 38]. The majority of patients (88.6%) had 6-8 cycles of paclitaxel/docetaxel plus carboplatin/cisplatin postoperatively, and 11.5% of patients received adjuvant chemotherapy of cyclophosphamide, bleomycin plus carboplatin before paclitaxel was available in China (Table 1).

### Histology image analysis and validation

H&E histological sections of multiple metastases from paraffin-embedded tumor blocks were generated, digitalized (Aperio, 20x, 0.5μm/pixel) and analyzed using open source R package CRImage [53] (Figure 1). The cell classifier was trained based on 100 features comprised of mostly morphological and some textural measurements as described in [25]. Cells were classified based on their morphological differences of the nucleus positive for haematoxylin stain without using immunohistochemical target stains. Immune cells typically display small, round and homogeneously basophilic nuclei; cancer cells in general have nuclei of larger size and greater variability in texture and shape. Both cell types can be differentiated from stromal cells such as fibroblasts, myofibroblasts, fibrocytes, vascular pericytes and endothelial cells that contain more elongated nuclei. The average amount of cells classified in each tumor section was 362,417 (interquartile range 208,129-502,789) for cancer cells, 92,861 (interquartile range 39,075-106,220) for stromal cells and 67,433 (interquartile range 27,944-90,852) for lymphocytes.

Validation of the classifier on tumors other than those from the ovary was performed using two approaches. First, a total of 4,633 single-cell annotations from two pathologists were collected from all six sites (cancer = 2,668, lymphocyte = 1,120, stromal = 845; Figure 2a, Supplementary Figure 6). Note that this data was only used for testing and not for training the classifier. Subsequently, sensitivity and specificity as well as the balanced average of both were computed (Figure 2b, Supplementary Figure 7 and 8). The second approach was performed by comparing proportions of cancer cells, lymphocytes and stromal cells to total amount of cells in 24 randomly selected fields of view. The fields of view were 2000 × 2000 square pixel images at a resolution of 0.5μm per pixel, representing 6 different metastasis sites each with four fields of view. Automated image analysis results were benchmarked against scores from the pathologists (DNR, KK) using Pearson's correlation test.

### Tumor microenvironmental diversity measures

We quantified the ecosystem diversity for a tumor *j* using the Shannon diversity index [45]:

Hj=−∑i=1Rpjilogpji' Eq.1

where *p_ij_* is the proportion of the *i* th cell type in tumor *j*, and *R* is the number of different cell types. A high value of the Shannon diversity index represents a heterogeneous environment populated by similar proportions of different cell types, whilst a low value indicates a homogeneous ecological composition. A known disadvantage of Shannon diversity is that it only accounts for species abundance and not absence [42]. We therefore also compared it with the Simpson diversity index [46]. The Simpson index of tumor *j* was defined as follows:

Dj=∑iRpji'2 Eq.2

where *p_ij_* is the proportion of the *i* th cell type in tumor *j* and *R* the number of different cell types.

### Other statistical methods

Survival analysis was carried out using R. Survival curves were plotted according to Kaplan-Meier [21] method and the difference in survival was assessed using the log-rank test. Cox proportional hazards model [13] was used to identify prognostic factors in univariate and multivariate analyses. To be considered for multivariate analysis, a factor had to be statistically significant (*p* < 0.05) in univariate analysis. Continuous variables were dichotomized due to their skewed distribution (skewness ranging between −0.4 and 1) by employing either an iterative thresholding approach or if no optimal threshold could be found by dividing patients into two equal-size groups based on the median. Lymphocyte ratio was dichotomized into a group of lower 33% and a group of higher 67%, stromal ratio into a group of lower 50% and a group of higher 50%, both in line with our previous study [25]. To evaluate the proposed uni/multivariate survival models, progressively decreasing number of samples from 100% to 70% were randomly drawn 1000 times from the cohort without replacement, survival analysis was performed and log-rank test p-value was recorded. This was used to test the stability of the uni/multivariate survival model by computing the percentage of times where it remained statistically significant. Kruskal-Wallis test was used for testing association between a continuous variable and a categorical variable. Fisher's exact test was used to test for association between categorical variables. Cell ratio was measured as the ratio of a cell type to all cells in a tumor. Variability of scores was measured using standard deviation. For testing the amount of tissue required for our analysis, we divided each section image into two halves horizontally or vertically, thereby obtaining equal sized tissue areas. New scores were computed using half of the tissue and compared with the original scores using absolute difference.

### Data and software availability

R code (Sweave) and data files to reproduce the reported results are available as supplementary data on www.yuanlab.org.

## SUPPLEMENTARY MATERIALS FIGURES


