# Supplementary material for: Similarity and diversity of the tumor microenvironment in multiple metastases: critical implications for overall and progression-free survival of high-grade serous ovarian cancer
Source: Oncotarget. 2016 Sep 19;7(44):71123–35. doi: 10.18632/oncotarget.12106 (PMC5342067; doi:10.18632/oncotarget.12106)
Supplement: Supplementary file 1 [file oncotarget-07-71123-s001.pdf]

Similarity and diversity of the tumor microenvironment in multiple metastases: critical implications for overall and progression-free survival of high-grade serous ovarian cancer

Supplementary Material

Supplementary Figure 1

Survival plots with 95% confidence interval

Supplementary Figure 2

Shannon diversity distribution in different metastasis sites and average cell type percentage per site

Supplementary Figure 3

Comparison of Simpson diversity with Shannon diversity

Supplementary Figure 4

MetDiv score comparison between presence/absence of omentum, peritoneum, lymph node or appendix metastasis

Supplementary Figure 5

Random sampling cells from metastasis samples

Supplementary Figure 6

Distribution of cell types annotated at each tumor site.

Supplementary Figure 7

Sensitivity of the cell classifier

Supplementary Figure 8

Specificity of the cell classifier

Supplementary Figure 1

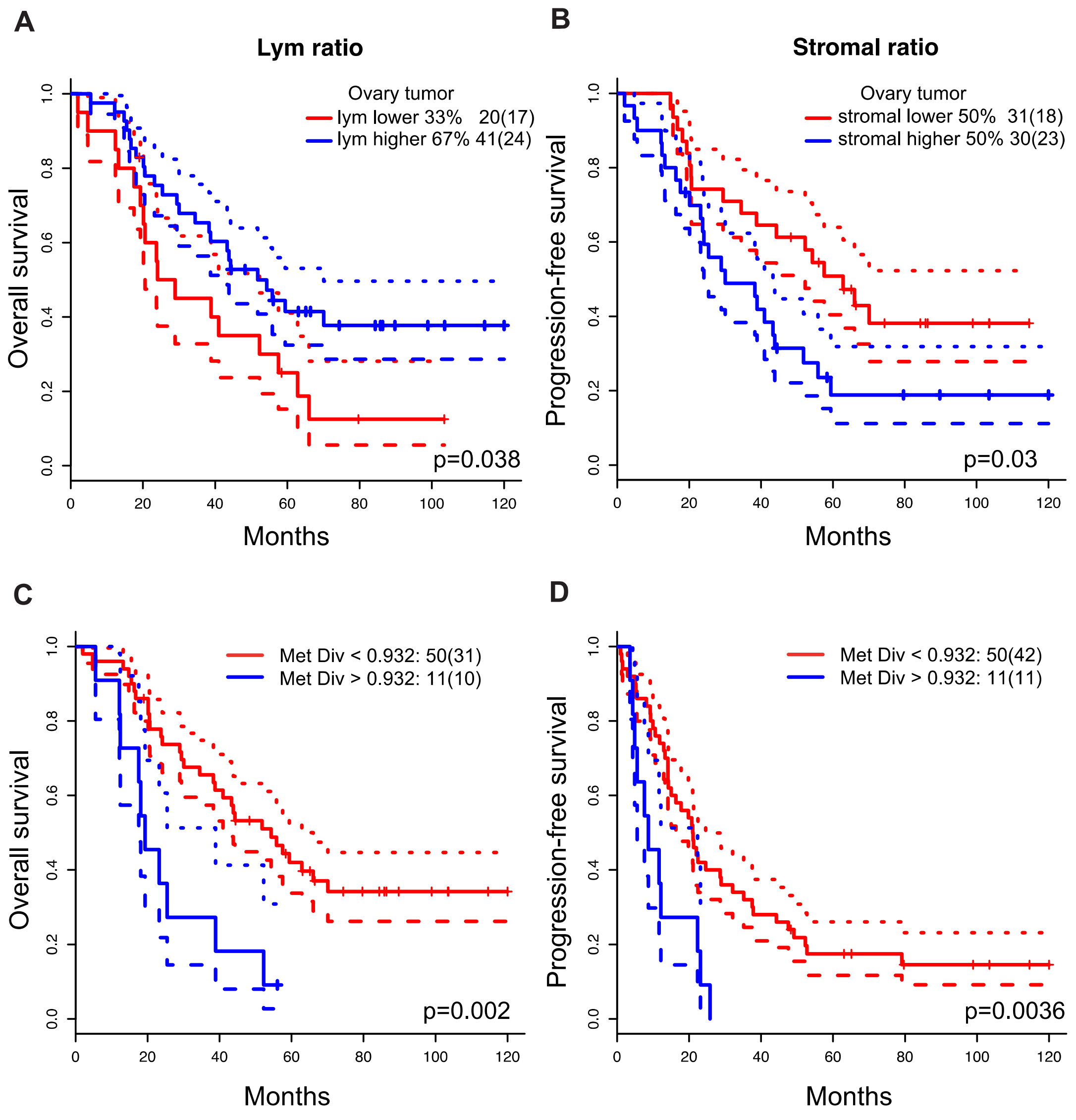

Supplementary Fig 1. Survival plots with confidence intervals. A. Overall survival stratified by lymphocyte ratio of the ovary tumor (red: lym ratio<0.07; blue: lym ratio≥0.07). B. Overall survival stratified by stromal ratio of the ovary tumor (red: stromal ratio<0.24, blue: stromal ratio≥0.24). C. and D. Kaplan-Meier survival curves to illustrate duration of OS and PFS for patients with a low (red) or high (blue) MetDiv score. The 95% confidence interval for all plots is illustrated via dashed and dotted lines.

Supplementary Figure 2

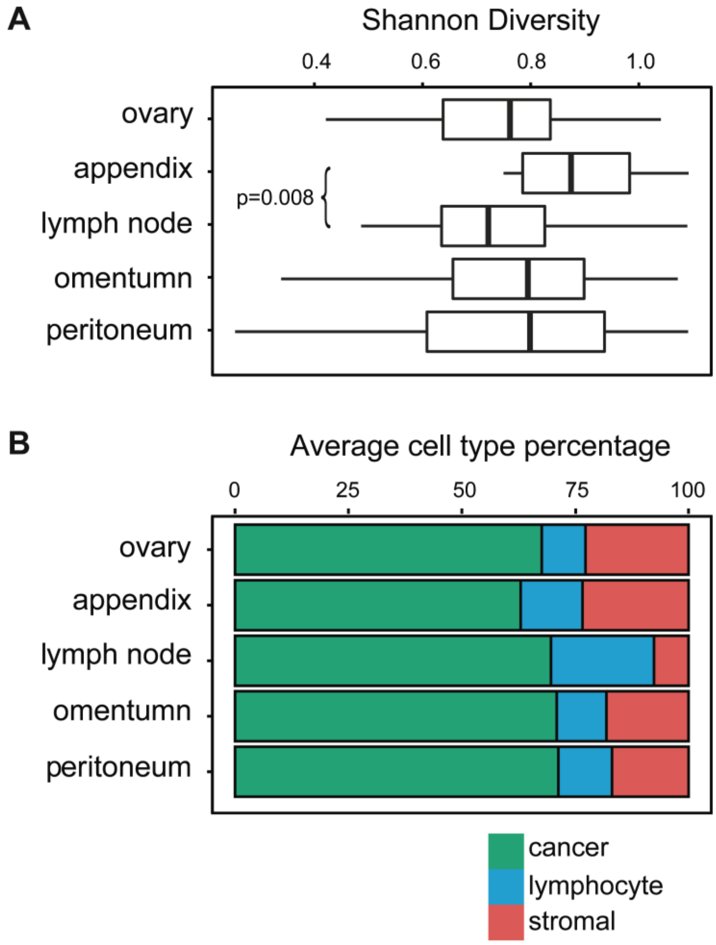

Supplementary Fig 2. Diversity distribution and average cell type percentage. A. Boxplot showing the Shannon diversity of ovarian tumors and different metastasis sites. Spleen (n=1) and umbilicus (n=2) were not included due to the small sample size. B. Barplot illustrating the cell composition of the ovarian tumors and metastases given in A.

Supplementary Figure 3

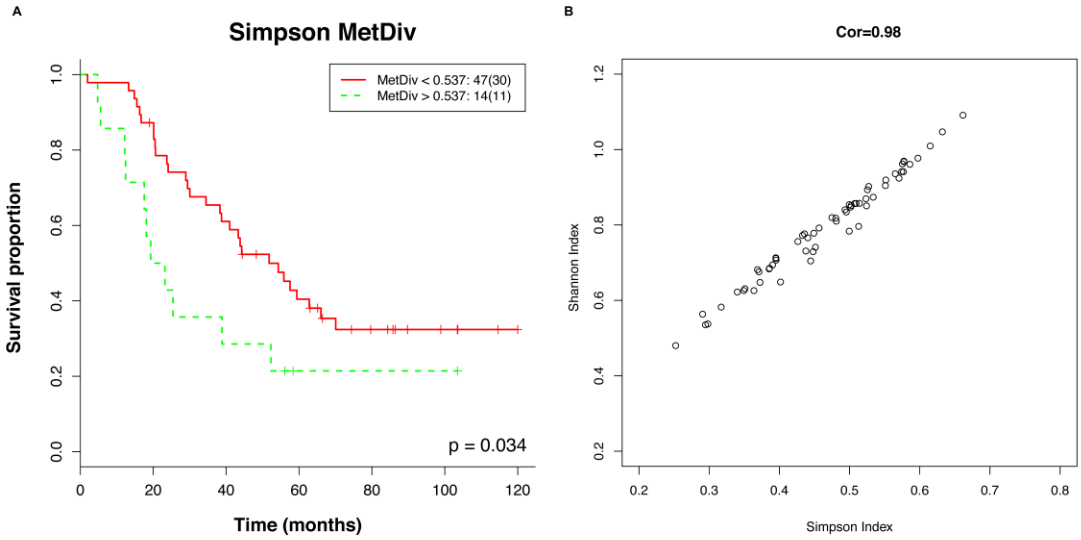

Supplementary Fig 3. Comparison of Simpson diversity with Shannon diversity. A. Kaplan-Meier survival curves for OS using Simpson diversity index to dichotomize patients. B. Pearson correlation analysis of Simpson diversity and Shannon diversity.

Supplementary Figure 4

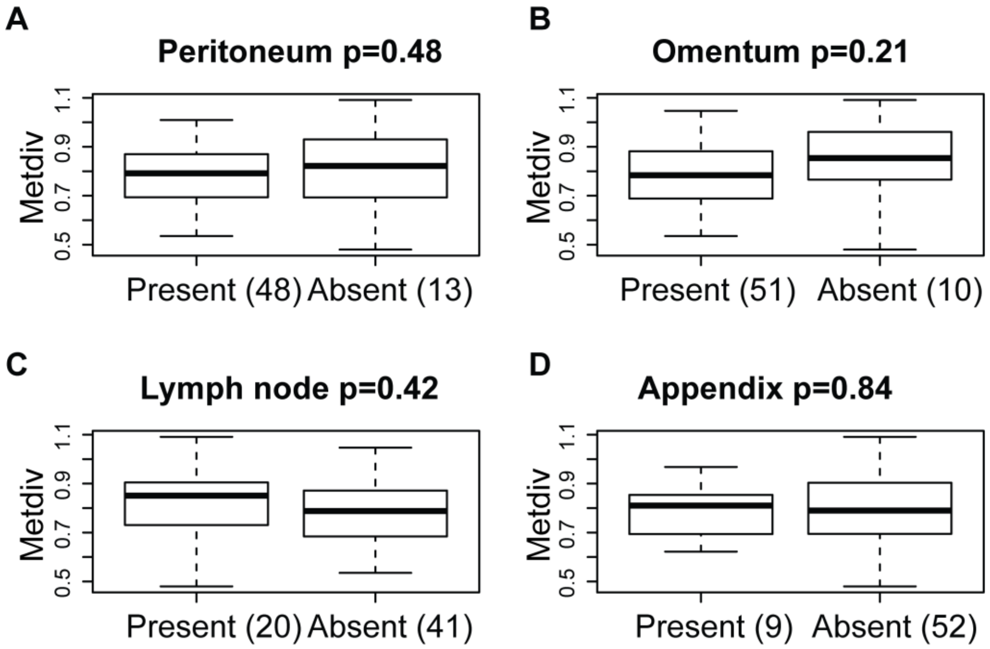

Supplementary Fig 4. MetDiv score comparison. A. - D. Presence/ absence of peritoneum/omentum/lymph node/appendix metastasis.

Supplementary Figure 5

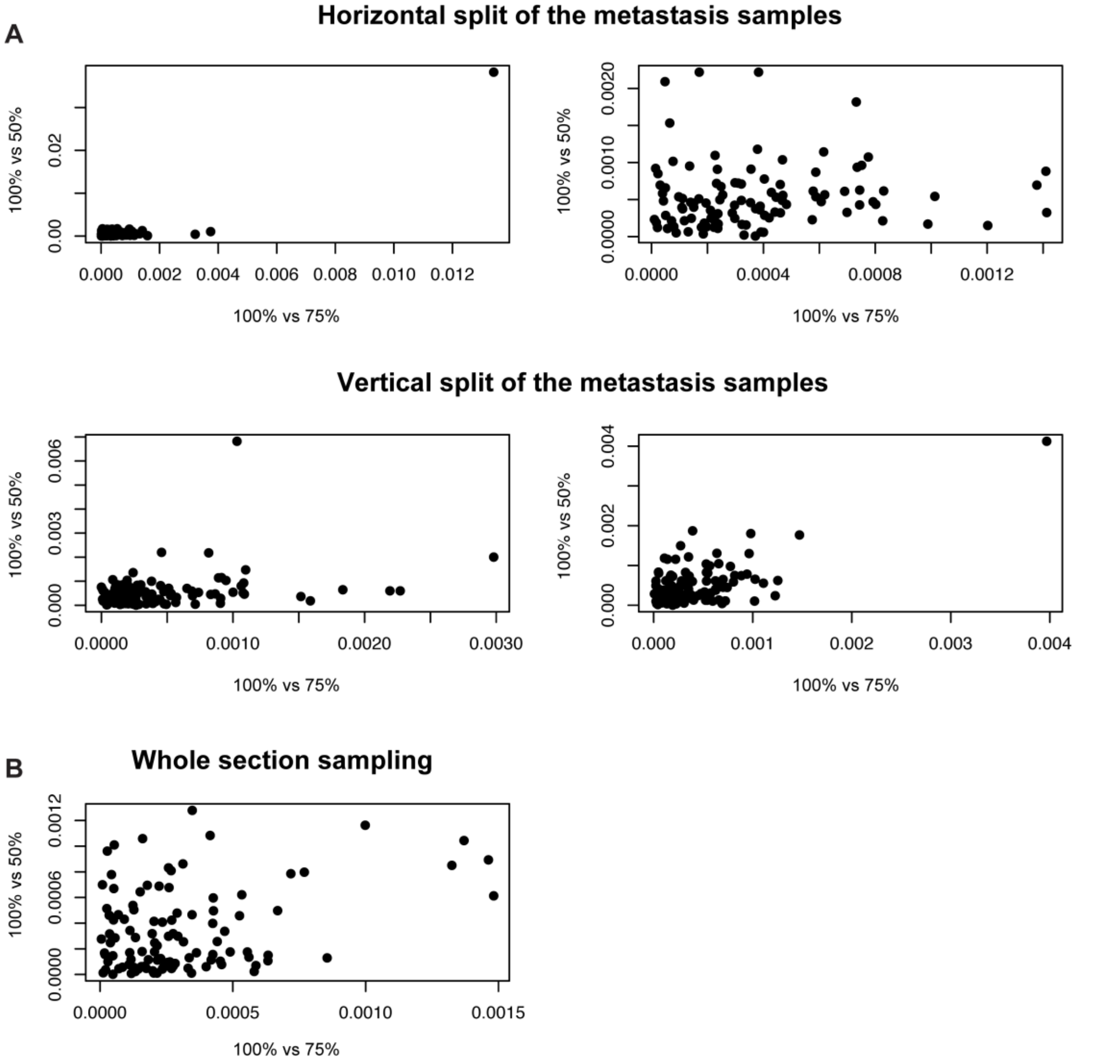

Supplementary Fig 5. Sampling cells with replacement from metastases. Scatter plots showing absolute differences between the whole-section scores 100% versus 75% slide scores and 100% versus 50% scores. Each metastasis is depicted as a point. A. Result of sampling cells from either half of the section after horizontal and vertical splitting. B. Sampling cells from the whole section without splitting.

Supplementary Figure 6

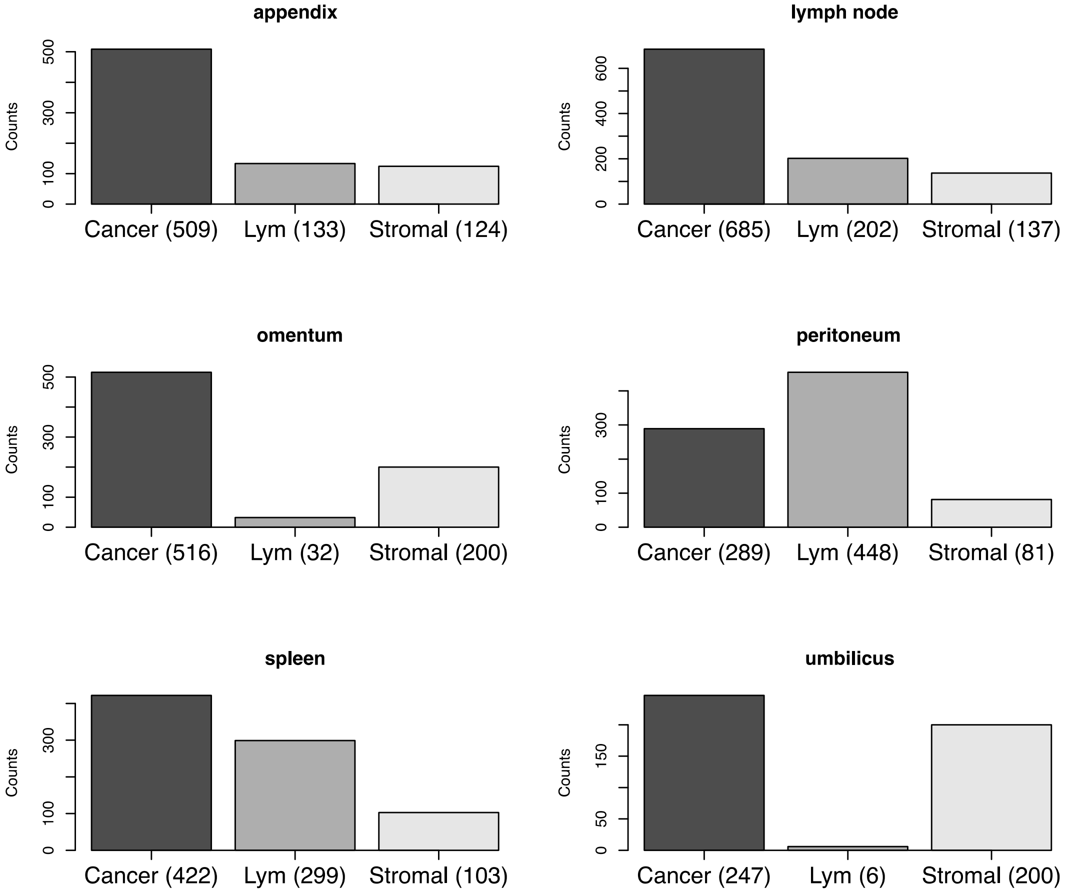

Supplementary Fig 6. Distribution of cell types annotated at each tumor site. Cancer cells are shown in black, lymphocytes in dark gray and stromal cells in light gray.

Supplementary Figure 7

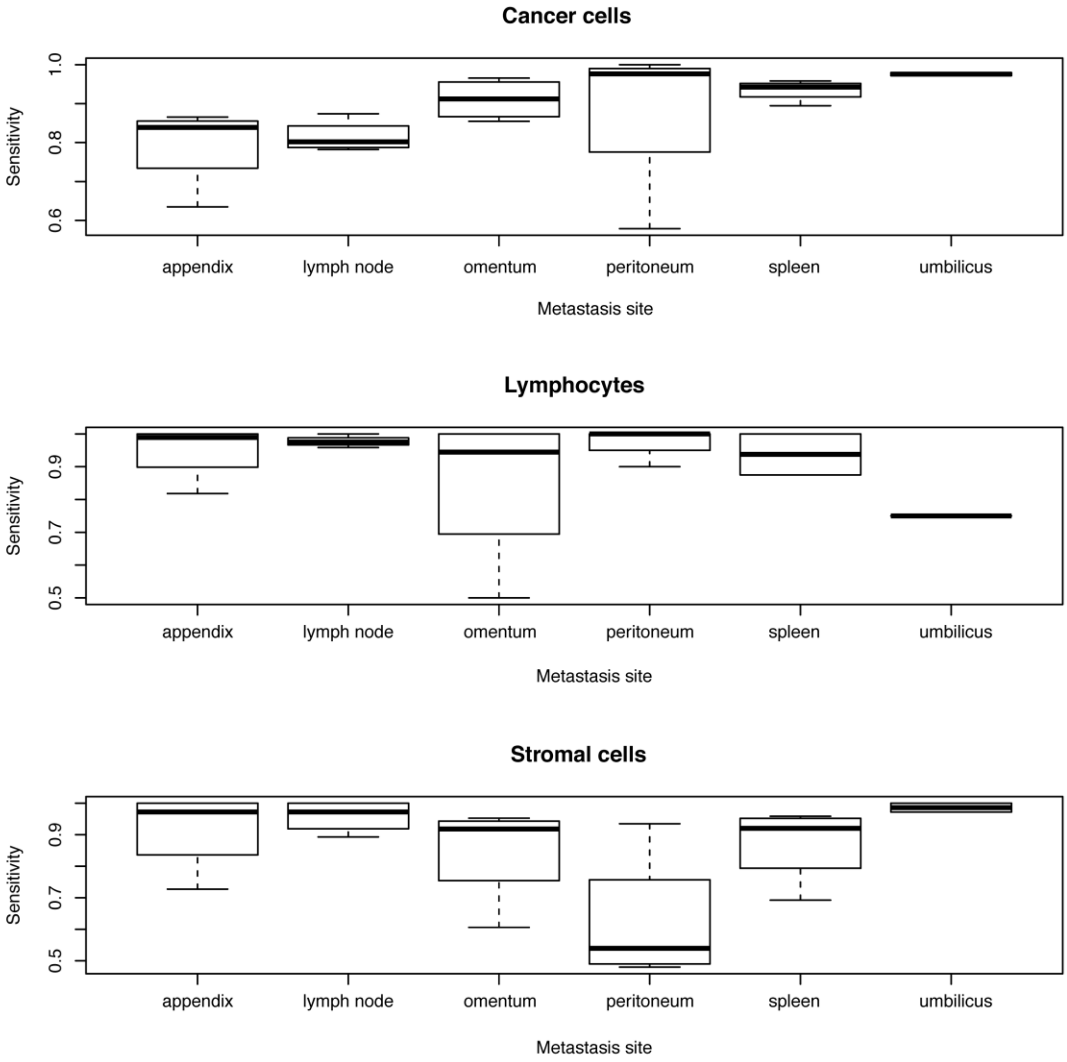

Supplementary Fig 7. Sensitivity of the cell classifier. Boxplot depicting the sensitivity of the automated cell detection for cancer cells, lymphocytes and stromal cells in all local metastasis sites.

Supplementary Figure 8

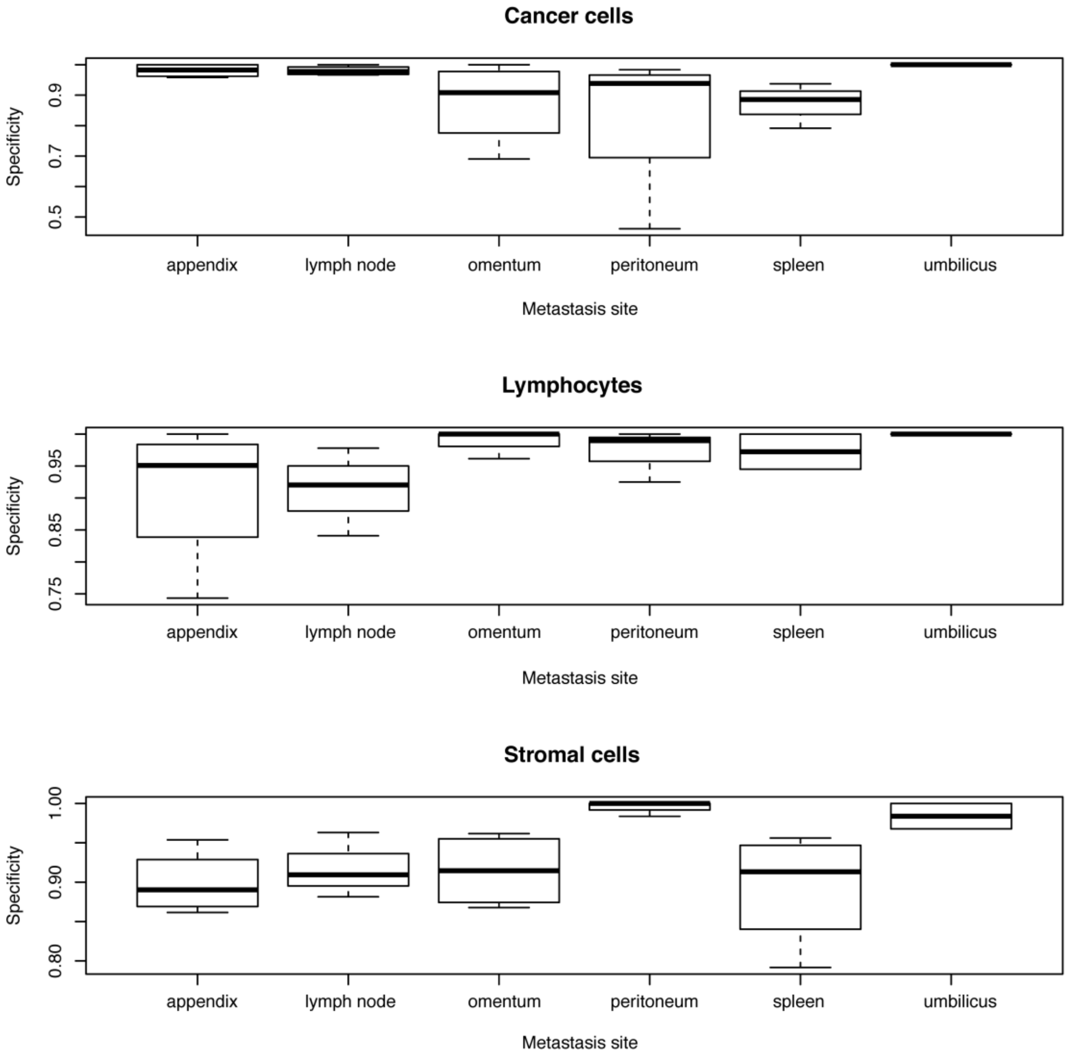

Supplementary Fig 8. Specificity of the cell classifier. Specificity of the automated cell detection for cancer cells, lymphocytes and stromal cells in all local metastasis sites.
